# Variation in foraging activity influences area-restricted search behaviour by bottlenose dolphins

**DOI:** 10.1098/rsos.221613

**Published:** 2023-06-14

**Authors:** Oihane Fernandez-Betelu, Virginia Iorio-Merlo, Isla M. Graham, Barbara J. Cheney, Simone M. Prentice, Rachael Xi Cheng, Paul M. Thompson

**Affiliations:** ^1^ Lighthouse Field Station, School of Biological Sciences, University of Aberdeen, Lighthouse Field Station, Cromarty IV11 8YL, UK; ^2^ Leibniz Institute for Zoo and Wildlife Research (IZW), Berlin 10315, Germany

**Keywords:** area-restricted search behaviour, echolocation buzzes, bray calls, bottlenose dolphins, machine learning, passive acoustics

## Abstract

Area-restricted search (ARS) behaviour is commonly used to characterize spatio-temporal variation in foraging activity of predators, but evidence of the drivers underlying this behaviour in marine systems is sparse. Advances in underwater sound recording techniques and automated processing of acoustic data now provide opportunities to investigate these questions where species use different vocalizations when encountering prey. Here, we used passive acoustics to investigate drivers of ARS behaviour in a population of dolphins and determined if residency in key foraging areas increased following encounters with prey. Analyses were based on two independent proxies of foraging: echolocation buzzes (widely used as foraging proxies) and bray calls (vocalizations linked to salmon predation attempts). Echolocation buzzes were extracted from echolocation data loggers and bray calls from broadband recordings by a convolutional neural network. We found a strong positive relationship between the duration of encounters and the frequency of both foraging proxies, supporting the theory that bottlenose dolphins engage in ARS behaviour in response to higher prey encounter rates. This study provides empirical evidence for one driver of ARS behaviour and demonstrates the potential for applying passive acoustic monitoring in combination with deep learning-based techniques to investigate the behaviour of vocal animals.

## 1. Introduction

Predators are expected to adjust their movements in response to prey distribution by remaining in areas rich in prey resources [1]. Increasing turning rates and decreasing displacement distance can be used to identify periods of area-restricted search (ARS) behaviour, which are likely to occur in response to higher prey availability [2]. Given the heterogeneous distribution of resources, predators that engage in ARS behaviour are predicted to increase their residency time in the vicinity of encountered prey [3,4]. For many marine predators, identification of ARS within tracks of tagged individuals has been critical for characterizing spatio-temporal variation in foraging activity [5,6]. However, ascertaining the drivers of ARS behaviour remains challenging in marine predators, due to the difficulties of empirically linking movement patterns to feeding events at sea [7–9].

For animals that use distinct vocalizations when searching for and encountering prey, drivers of ARS behaviour may be investigated using passive acoustic techniques [10]. Bottlenose dolphins produce many different vocalizations, several of which are directly linked to foraging [11]. For instance, like many other cetaceans, they produce echolocation buzzes, groups of echolocation clicks emitted at a high repetition rate, when they close in on prey [12]. To investigate the drivers of dolphin ARS behaviour, Bailey *et al*. [10] modelled patterns of echolocation clicks, based on recordings from single echolocation data loggers, and characterized the variation in dolphin occurrence in relation to the presence of echolocation buzzes. As predicted, the probability of dolphins leaving areas around each device decreased with a higher proportion of foraging activity early in the encounter. However, contrary to expectations on ARS behaviour, animals were more likely to leave the area when there was a high proportion of foraging activity later in the encounter. To explain this inconsistency, Bailey *et al*. [10] hypothesized that the restricted range of single echolocation data loggers (less than 1000 m; Roberts & Read [13]) may not capture entire periods of ARS behaviour within each foraging patch. Further, to correct the underestimation of foraging activity from single acoustic recorders caused by the directional nature of echolocation clicks [14], Bailey *et al*. [10] based their results on modelled echolocation buzzes instead of vocalizations detected by the recorders. Therefore, omnidirectional cues for quantifying encounters with prey, coupled with broader arrays of passive acoustic devices, may provide more powerful tests of this hypothesis.

In addition to echolocation buzzes, bottlenose dolphins produce bray calls, low-frequency (less than 2 kHz) omnidirectional vocalizations specifically associated with attempts to capture salmonids [15]. Echolocation data loggers can be deployed for periods of several months and the resulting data routinely processed to identify periods of occurrence and foraging activity [16,17]. However, the collection and analysis of long-term data on variation in broadband vocalizations, such as bray calls, have been constrained both by the capacity and longevity of underwater sound recorders and the need for manual analysis [18,19]. These constraints are now being overcome by the availability of relatively low-cost archival sound recorders [20,21] and the development of automatic detectors based on deep learning techniques [22–24].

Here, we build upon the approach by Bailey *et al*. [10] to investigate drivers of ARS behaviour in another population of bottlenose dolphins, by testing whether their residency time increased in response to foraging. Instead of single acoustic devices, we deployed arrays of echolocation data loggers and broadband sound recorders to characterize both occurrence and foraging activity within two known foraging areas [25]. We used two different proxies for foraging: (i) echolocation buzzes, identified by modelling echolocation inter-click intervals (ICIs) [17] and (ii) bray calls, automatically detected using deep learning techniques, building upon the methodology of Bergler *et al*. [22]. We hypothesised that dolphins would remain longer within each of these foraging areas when the detection rates of foraging proxies within the encounter increased.

## 2. Material and methods

### 2.1. Study area

**Figure 1.**
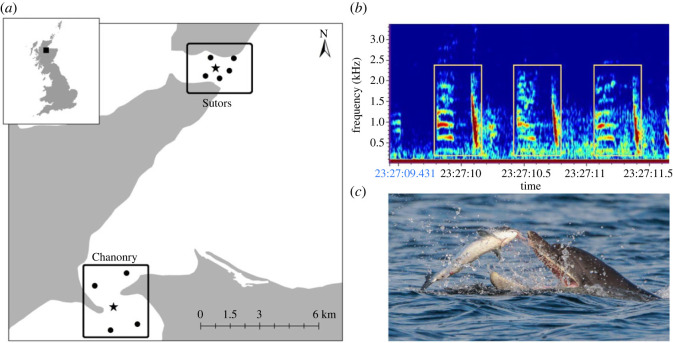
(*a*) Map showing the passive acoustic monitoring arrays deployed at Sutors and Chanonry in NE Scotland including CPOD (circles (●)) and CPOD + SoundTrap (stars (★)) locations. (*b*) Example of spectrogram (Raven Pro 1.6) with three bray calls annotated with yellow boxes. (*c*) Bottlenose dolphin feeding on Atlantic salmon at Sutors. Photo by Dr B. Cheney - © Lighthouse Field Station, University of Aberdeen.

This study was conducted in two narrow channels within the Moray Firth Special Area of Conservation (SAC), NE Scotland: Sutors (57° 41.41′N, 03° 59.18′W) and Chanonry (57° 5.14′N, 04° 5.85′W; figure 1). During summer months, these two channels are intensively used by the resident population of bottlenose dolphins that occurs along the east coast of Scotland [26–28]. Visual observations have previously shown that both Sutors and Chanonry narrows are foraging hotspots [25] where dolphins exhibit ARS behaviour [29].

### 2.2. Acoustic deployments

Between May and September 2018, echolocation data loggers (CPODs*;* Chelonia Ltd, UK) were deployed 2 m above the seabed to record continuously at six sites within Sutors and five sites within Chanonry (figure 1). In Sutors, the highest concentration of bottlenose dolphin sightings occurs at the eastern entrance of the channel [27,30]. In Chanonry, dolphin density is higher in the northern part [31], but foraging behaviour occurs in a relatively small area (0.3 km radius) next to the western promontory of the channel [29]. Arrays of CPOD devices were designed taking account of the known distribution of dolphins in each of the sites, to maximize the detection of echolocation clicks.

Between June and September, a single broadband recorder (SoundTrap ST300HF; Ocean Instruments, NZ) was also deployed at the mooring in the middle of each array and was duty cycled to record 50% of the time (10 min every 20 min) at a sampling rate of 48 kHz.

### 2.3. Encounter definition

We used the manufacturer's custom software (www.chelonia.co.uk) to extract echolocation click detections from each of the CPODs. Following the manufacturer's guidelines, we only included dolphin clicks classified as high and moderate quality. Unique dolphin encounters within each site were defined based on the interval between echolocation clicks detected by each array of CPODs. Specifically, we defined a dolphin encounter as a group of echolocation clicks, detected by any CPOD in the array, containing no gaps longer than 10 min [32–36].

### 2.4. Detection of foraging behaviour

After grouping echolocation click detections by CPOD array, echolocation buzzes were identified by modelling the ICI with a Gaussian mixture model [17]. ICIs can be categorized into three groups: ‘buzz ICIs’, echolocation buzzes linked to foraging activity [12]; ‘regular ICIs’, corresponding to the known ICIs of regular click trains [37]; and ‘long ICIs’, corresponding to ICIs between different click trains. To assign each ICI to one of those three processes, we fitted a Gaussian mixture model to the log-transformed ICI, setting the number of component distributions *k* to be equal to three. The results of the model were used to allocate each ICI into one of the three ICI groups and identify echolocation buzzes.

Bray calls were identified using DOLPHIN-SPOT, a deep convolutional neural network (CNN)-based bray detector following the methodology of Bergler *et al*. [22]. DOLPHIN-SPOT produces an output that divides audio recordings into segments of variable length and labels them as bray call positive or negative (details in the electronic supplementary material S1—DOLPHIN-SPOT).

### 2.5. Statistical analysis

To investigate whether dolphins remained longer in the area when foraging activity increased, we modelled dolphin encounter duration (decimal minutes) as a function of the proportion of foraging positive minutes within the encounter. Foraging positive minutes were defined using two metrics. First, as minutes with more than five echolocation buzzes and, second, as minutes with at least one segment labelled as bray call positive. Since tides affect the occurrence of dolphins in Chanonry but not in Sutors [36], channel (Sutors/Chanonry) and tidal stage in mid-encounter (Flood-High-Ebb-Low) were also included as explanatory variables. We used generalized linear models, with a gamma distribution. The link function was chosen between the identity and log link functions based on the lowest Akaike information criterion (AIC; [38]). Due to differences in CPOD and SoundTrap deployment durations, two datasets were created for echolocation buzzes and bray calls respectively, and they were analysed in separate models. Models were assessed following a stepwise exclusion of variables using the variation in AIC (*Δ*AIC), starting from the models including all explanatory variables and all possible interactions. The model with the lowest AIC value was considered the most parsimonious and best approximating model [39]. We checked autocorrelation in residuals using autocorrelation plots (ACF). To match the SoundTrap 10 min duty cycle, only encounters longer than 10 min were retained in the echolocation buzz dataset. Similarly, the bray call dataset only considered dolphin encounters with at least 10 min of SoundTrap recording.

We used the statistical program R v.4.1.2 in all the analyses [40]. Model assumptions were verified through visual inspection of the residual plots [41] and using the R package *performance* [42].

## 3. Results

Dolphin vocalizations were detected every day throughout the 17-week study period for an average of 5.3 h/day at Sutors and 3.6 h/day at Chanonry.

The Gaussian model with three components led to the identification of the three ICI groups, including buzz ICIs (electronic supplementary material, figure S2.1 in the electronic supplementary material S2). The mean buzz ICI was 4.5 ms (s.d. ± 2.3 ms). Of the 2747 dolphin encounters recorded by CPODs, 1444 were longer than 10 min and 718 contained more than 10 min of SoundTrap broadband recordings (table 1). In both datasets, encounters were significantly shorter at Chanonry than at Sutors (Echolocation buzz dataset: Kruskal–Wallis *X*^2^ = 43.3, d.f. = 1, *p* < 0.001; bray call dataset: Kruskal–Wallis *X*^2^ = 22.7, d.f. = 1, *p* < 0.001; table 1). No obvious seasonal trend in the duration of dolphin encounters was observed in either of the channels (electronic supplementary material, figure S2.2 in the electronic supplementary material S2).

**Table 1.** Number of bottlenose dolphin encounters and median encounter duration in decimal minutes, including upper and lower quartiles.

		total encounters	median encounter duration (lower–upper quartile)	total encounters without foraging positive minutes (median encounter duration)	total encounters with foraging positive minutes (median encounter duration)
echolocation buzz dataset	*Sutors*	816	33.0 min (19.3–55.8 min)	18 (14.5 min)	798 (33.6 min)
	*Chanonry*	628	24.3 min (15.7–41.7 min)	46 (14.5 min)	582 (25.8 min)
bray call dataset	*Sutors*	429	38.5 min (24.7–61.2 min)	118 (25.3 min)	311 (46.2 min)
	*Chanonry*	289	29.5 min (20.2–47.9 min)	69 (20.5 min)	220 (31.9 min)

Trained on 14% of the dataset, the CNN model was validated on 20 10 min unseen raw audio files where it detected bray calls with an accuracy of 98.7%, precision of 88.5% and false positive rate of 1% (see details in the electronic supplementary material S1). This model was then applied to the entire 2436 h of broadband audio dataset, where it automatically identified 10 348 min that contained at least one bray call positive segment (7% of the complete broadband dataset).

**Figure 2.**
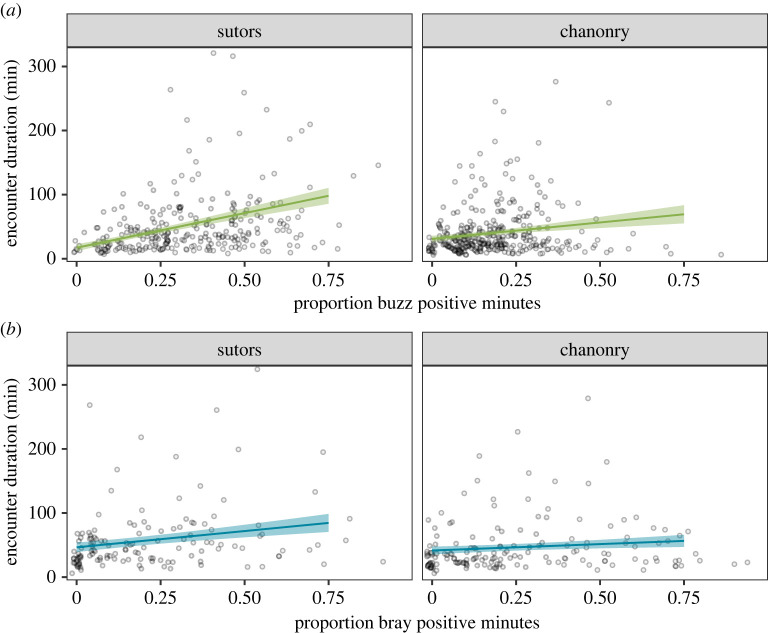
Predicted dolphin encounter duration (minutes) in relation to the proportion of foraging positive minutes in the encounter for (*a*) echolocation buzzes and (*b*) bray calls divided by channel (Sutors: left; Chanonry: right) during flood. Shaded areas are the 95% confidence intervals. Plots include raw data (grey points).

As predicted from our hypothesis, dolphin encounters were longer when the proportion of foraging positive minutes was greater within the encounter. A positive trend was observed for both echolocation buzzes (figure 2*a*) and bray calls (figure 2*b*). The trend was weaker for bray calls than for echolocation buzzes and, overall, weaker in Chanonry compared to Sutors. For the echolocation buzz dataset, the most parsimonious model retained the interaction between (i) proportion of foraging positive minutes and channel (chisq = 16.8, d.f. = 1, *p* < 0.001), (ii) proportion of foraging positive minutes and tidal stage (chisq = 12.2, d.f. = 3, *p* < 0.01) and (iii) tidal stage and channel (chisq = 20.4, d.f. = 3, *p* < 0.001). This relationship was similar for a restricted dataset that used only those data for a subsample of encounters that contained echolocation clicks with higher sound pressure levels, representing encounters that were likely to be closer to the receivers (electronic supplementary material S3). For the bray call dataset, the most parsimonious model retained the interaction between (i) foraging positive minutes and channel (chisq = 3.9, d.f. = 1, *p* < 0.05) and (ii) tidal stage and channel (chisq = 9.7, d.f. = 3, *p* < 0.05; electronic supplementary material S2).

## 4. Discussion

ARS behaviour in marine predators is widely used as a proxy for encounters with heterogeneously distributed prey, however, empirical evidence for this relationship is sparse [10,43,44]. Previous visual tracking at one of our study sites demonstrated that surface-feeding events were more likely within areas that dolphins searched intensively [29]. Here, we showed that two different acoustic proxies for foraging were associated with longer bottlenose dolphin encounter duration within the two study sites.

These results further support the theory that predators engage in ARS behaviour by increasing their residency time in an area after encountering prey [2,3,45]. In this study, prey encounters were inferred by using the proportion of foraging positive minutes to link the encounter with the presence of prey. Previously, Bailey *et al*. [10] found evidence of dolphins displaying ARS behaviour in response to foraging that occurred during the first third of an encounter but, unlike this study, not when considering foraging occurrence across the whole encounter. To overcome one of the limitations of Bailey *et al*.'s [10] study, who deployed single recorders at four offshore study sites, we used arrays of recorders within two constrained (less than 6 km^2^) coastal areas where dolphins are known to forage regularly [25,29]. Although both studies used the detections of echolocation buzzes to infer foraging behaviour, here these were obtained through combined detections across each acoustic array, increasing the effective sampling area and the probability of detecting both echolocation buzzes and the full extent of the ARS behaviour. Differences in the results between these two studies may, therefore, have been caused by variation in design.

Bottlenose dolphins are selective opportunistic predators that feed on a variety of prey species, of varying quality [46]. Echolocation buzzes have not been linked to a specific prey and thus may represent encounters with various species. In contrast, to date, bray calls have only been associated with salmonid prey [15,19]. Salmonids are among the largest prey species of these dolphins [47] and are known to influence behavioural patterns of these and other coastal marine mammals at a variety of scales [48].

The increase in dolphin encounter duration in relation to a higher proportion of bray positive minutes may be biased by the mechanics of capturing salmon. Due to the size of salmon, dolphins in the area are often seen regurgitating these fish repeatedly before swallowing them (B. Cheney, personal observation), effectively increasing dolphin prey handling time. Therefore, dolphin encounters may be longer in response to successful feeding events. However, we could not disentangle the relative contribution of prey handling time to the observed increase in encounter duration because the average handling time for dolphins foraging on salmon is unknown. Further, the proportion of foraging minutes within each encounter may also be influenced by prey density within a patch and prey depletion rate [49,50]. Theory assumes that predators remain in a prey-rich patch until the density of prey decreases up to the point where the area is not profitable [51]. Here, we showed that a high proportion of foraging positive minutes was linked to longer encounters, demonstrating that dolphins remain in prey-rich areas. Although the relationship between bottlenose dolphin foraging call production rate and prey density is unknown, other predators have been found to adjust their behaviour to the density of their prey [50,52]. Future studies could investigate the rate of production of foraging calls within encounters to explore whether a decrease in foraging calls leads to patch departure.

Our analyses showed a weaker correlation between encounter duration and foraging activity using bray calls than using echolocation buzzes (figure 2). This is in contrast with the results found by Weimerskirch *et al*. [9], where predators engaged in ARS behaviour only after capturing large prey items. Predator satiation is directly linked to prey size, and the level of satiation plays a major role in the behaviour of predators [53]. Therefore, one possible explanation for the dissimilarity between our study and the findings of Weimerskirch *et al*. [9] is that dolphins may become satiated more quickly predating on salmonids than on smaller prey, leaving the area earlier. This weaker correlation between encounter duration and foraging using bray calls compared to echolocation buzzes could also be attributed to the differences in the methodology, data availability and detection probabilities of different call types. First, differences between these two vocalizations could be related to different detection ranges. CPODs detect echolocation clicks up to 1 km away [13], but detection probabilities for buzzes may be lower for more distant groups [54,55]. We used pooled detections from an array of CPODs across each foraging area (figure 1) and also demonstrated similar relationships when using a subset of data from encounters that were likely to be closer to CPODs (electronic supplementary material S3). Thus, while the stronger relationship for buzzes in figure 2 may partly be due to shorter, distant, encounters that have fewer detected buzzes, potential differences in detection probabilities do not appear to be driving this relationship. While there are no estimates of the detection range of bray calls, like any other low-frequency sound, this second proxy of foraging is more likely to be detected at greater distances. Although we increased the cumulative CPOD detection range by deploying arrays of devices, it is therefore possible that the SoundTraps recorded some bray calls from dolphins located out of the detection range of the CPOD arrays. In addition, the weaker correlation between encounter duration and foraging using bray calls could have been caused by the duty cycling of broadband recorders. Our CPODs recorded continuously, but SoundTraps were duty cycled to record 50% of the time, resulting in samples sizes for bray calls being smaller and biased towards longer encounters (table 1). Further studies with continuous broadband recordings made at higher sampling rates could be integrated with visual observations to explore how inferences from these two proxies for foraging may be influenced by different detection probabilities.

Overall, we found a positive correlation between encounter duration and proportion of time foraging within the encounter at both study sites, although the correlation was weaker in Chanonry than in Sutors (figure 2). In coastal areas, the tidal cycle has a major effect on the distribution and behaviour of prey, which can shape predator foraging behaviour [56–59]. While dolphin occurrence has not previously been linked to any tidal stage in Sutors, their occurrence increases during flood in Chanonry, and it has been hypothesized that this pattern may be related to cyclical changes in prey catchability, abundance or behaviour [36]. One explanation for the weaker link between foraging activity and encounter duration in Chanonry is that predator–prey interactions may be associated with specific phases of the tidal cycle at this site. Further research to investigate variation in prey fields throughout the tidal cycle in the area would be required to test this hypothesis.

Similarly to previous passive acoustic studies, notably Bailey *et al*. [10], our study used acoustic detections of foraging calls that could not be localized. These methods constrained our ability to study individual dolphin behaviour and thus our results represent dolphin groups. Furthermore, group size has the potential to influence both the proportion of foraging positive minutes and the duration of encounters. However, lack of localization and site- and context-dependent changes in dolphin vocalization rate limit the ability to infer group size from acoustic detections [60,61]. Arguably, dolphin encounters with a higher number of individuals could lead to higher vocalization rates. However, a study on Heaviside's dolphin (*Cephalorhynchus heavisidii*) found that dolphin echolocation buzz rate decreased with group size [62]. Geographical differences in the vocalization rate of bottlenose dolphins linked to group size have also been found [60] and site-specific information on our study population is lacking. Further research is required to investigate the link between dolphin vocalization rate and group size and their effect on encounter duration at these two sites. Nevertheless, in this study, we showed that two different proxies for predator foraging behaviour led to similar conclusions about predator ARS behaviour.

Higher endurance broadband recorders are now available, opening the potential to use new automated pattern recognition techniques to routinely extract distinct animal vocalizations from continuous long-term recordings [63,64]. These, in turn, could be used to explore other aspects of foraging theory, including investigating how patterns of bray call production within encounters affect decisions over when to leave prey patches [51]. Furthermore, continuous long-term recordings could also be used to test whether foraging decisions are moderated by other factors that may affect the length of dolphin encounters, such as disturbance from boat traffic [65] or other anthropogenic stressors.

## Data Availability

The datasets and R Code supporting this study are available from the Dryad Digital Repository: https://doi.org/10.5061/dryad.djh9w0w1n [66]. We included extra information, analyses and results in the supplementary material [67].
